# The research crisis in American institutions of complementary and integrative health: one proposed solution for chiropractic profession

**DOI:** 10.1186/s12998-019-0251-1

**Published:** 2019-06-17

**Authors:** Ian D. Coulter, Patricia M. Herman

**Affiliations:** 1 RAND Corporation, 1776 Main Street, P.O. Box 2138, Santa Monica, CA 90407-2138 USA; 2 UCLA, Los Angeles, CA USA; 3 Southern California University of Health Sciences, Whittier, CA USA

**Keywords:** Chiropractic research, complementary and integrative health research, research infrastructure, RAND Corporation

## Abstract

A crisis confronts the Complementary and Integrative Health (CIH) teaching institutions in the US. Research infrastructure is needed to build and sustain productive research programs and retain their own research faculty. In most health professions, this infrastructure is largely built through research grants. In CIH, most educational institutions are funded through student tuition, which has historically also had to be the source for building their research programs. Only a limited number of these institutions have emerged as National Institute of Health (NIH) grant-funded programs. As a result, the American chiropractic institutions have seen a retrenchment in the number of active research programs. In addition, although research training programs e.g., NIH’s K awards are available for CIH researchers, these programs generally result in these researchers leaving their institutions and depriving future CIH practitioners of the benefit of being trained in a culture of research.

One proposed solution is to leverage the substantial research infrastructure and long history of collaboration available at the RAND Corporation (https://www.rand.org) This article presents the proposed five components of the RAND Center for Collaborative CIH Research and the steps required to bring it to being: 1) the CIH Research Network – an online resource and collaborative site for CIH researchers; 2) the CIH Research Advisory Board – the governing body for the Center selected by its members; 3) the RAND CIH Interest Group – a group of RAND researchers with an interest in and who could provide support to CIH research; 4) CIH Researcher Training – access to existing RAND research training as well as the potential for the Center to provide a research training home for those with training grants; and 5) CIH RAND Partnership for Research – a mentorship program to support successful CIH research. By necessity the first step in the Center’s creation would be a meeting between the heads of interested CIH institutions to work out the details and to obtain buy-in.

The future success of CIH-directed research on CIH will require a pooling of talent and resources across institutions; something that the American chiropractic institutions have not yet been able to achieve. This article discusses one possible solution.

## Introduction

In their 2018 article, Adams et al. [1] drew attention to a crisis in what they termed a sustainable research culture in chiropractic. They state “At present however, there is not a mature research culture across the chiropractic profession largely due to deficiencies in research capacity and leadership, which may be caused by a lack of chiropractic teaching programs in major universities.” ([1], p1) In response they created the Chiropractic Academy for Research Leadership (CARL) whose purpose is to provide mentorship of successful early-career chiropractic researchers. This is an excellent program and deserves to be successful.

However, in the US, the problem is less of a lack of trained researchers than a lack of research infrastructure. Through their K awards, National Institute of Health (NIH) offers research training grants to individuals across all stages of their career, and the K awards ((https://www.nichd.nih.gov/grants-contracts/training-careers/extramural/career) and T32 awards (https://researchtraining.nih.gov/programs/training-grants/T32) offered by the National Center for Complementary and Integrative Health (NCCIH) particularly focus on training complementary and integrative health (CIH) researchers. Several chiropractors and others from the CIH community have benefited from such career awards. These awards require that the individuals are mentored. However what we are increasingly witnessing is that these successful scholars are either lost to the CIH institutions or find that their institutions lack the infrastructure to continue to advance or sustain their research programs and to successfully compete for grants. Many institutions also lack the kind of supporting culture that has been shown to be necessary for successful research programs and individuals [2, 3].

In most health professions, research infrastructure is largely built through research grants. Most CIH educational institutions are funded through student tuition, and historically this has also had to be the source for building their research programs. Few have emerged as NIH grant-funded research programs and those have largely benefited from NCCIH funding, which constitutes less than 1 % of the NIH budget. The number of American chiropractic institutions currently receiving grants is modest and given the increasing costs of sustaining research programs and recent fall in enrollments, there has been a substantial retrenchment in the number of active research programs.

We are left with the situation that at a time when there are more Doctors of Chiropractic with PhDs than at any time in chiropractic history, and while the profession can point to a cadre of active researchers, the chiropractic institutions are increasingly not the home for these scholars. In this article, we consider the possibility that perhaps an institution such as RAND could provide a supporting infrastructure and a supporting culture that could allow CIH researchers to be successful and remain at their institutions.

In previous work Herman and Coulter [4, 5] examined a related policy problem for the CIH professions: the fact that in many policy areas they are not treated as comprehensive healing professions but as modalities. We noted the importance research plays in framing the policies: “Research seems to be focused on the effectiveness of procedures and modalities and only rarely on the health care outcomes associated with receiving care from a member of a particular profession. Guidelines are based on research, and thus, it is not surprising that they also focus on procedures and modalities because research only focuses on modalities, not the professions.” ([4] p506) In medical trials it is common to compare a particular modality to usual and customary medical care which incorporates anything a medical practitioner would normally provide for that patient. So clearly a professional paradigm of care can be the subject matter of research without being reduced to a single modality. We would argue that is equally true for the CIH professions even given the variability in practices even within these professions.

While professions are characterized by having access to a body of knowledge and skills they are also characterized by producing knowledge, not simply consuming it. There are at least two major consequences for chiropractic by not participating in research. The first is that the research agenda will be defined by others resulting in research focusing on modalities, which reinforces the policies that treat the professions as modalities. The second is that its teaching institutions will be seen as technical teaching institutions and not as professional schools. This is particularly problematic in that recent years, chiropractic colleges have moved to becoming universities and have included that in their name. But universities are centers of advanced learning not just teaching institutions. They are by definition, research institutions. Those that teach there are expected to be scholars and those being taught expect to be taught by scholars.

In short, while having individuals doing chiropractic research is necessary but it is not sufficient. If it is being done outside of chiropractic institutions it weakens the institutions as centers of advanced learning. This is the challenge for the US chiropractic institutions.

## Methods

Although this is not intended as a methods paper we used several approaches to base our conclusions and to ground our recommendations. In our earlier study, we convened two separate expert panels [4, 5]. One panel included representatives from the CIH professions. The second included persons involved in making policy decisions with respect to the professions. Both identified research as a major area for concern. Secondly, both IDC and PMH are experienced reviewers for NCCIH, other NIH, and other funding organization’s research proposals and have seen first-hand the increasing disparity between the quality and sources of the submitted proposals. In the process of developing the proposal described below we have spoken widely to Presidents of 15 chiropractic and nine CIH other institutions and with other leaders in the CIH community and in a limited number of cases shared a written proposal. The discussions were both about the crisis in their research programs and the proposed RAND solution. We are currently planning a meeting at RAND later in September 2019 to launch the proposals with these stakeholders. RAND has undertaken to fund this initial meeting. To date we have found widespread consensus with our interpretation that there is a crisis in CIH research and the nature of that crisis. Although the focus in this paper is chiropractic, what we are proposing is for all the CIH.

## The RAND solution

The RAND Corporation is a nonprofit institution whose mission is to help policymakers make decisions that are based on the best available information. RAND has a tradition of objective high-quality research and analysis, has a long history of collaborating across diverse organizations, and has substantial research infrastructure. Leveraging this infrastructure might help address the CIH research crisis. We propose a comprehensive approach: a RAND Center for Collaborative CIH Research with the following five components.

1. CIH research network. This website will be the core of the Center and will include: a searchable directory of Center members and their research interests and experience; a list of resources including datasets and patient populations available; discussion group forums (e.g., message board, chat room) to share ideas and connect with others; webinars covering methods and new and ongoing research; calendar(s); posts on newly published research; lists and links to possible funding sources and Requests for Proposals; and links to other resources of interest. This online resource will be housed within RAND security and will be only open to members – although membership may be as simple as registration.
2. CIH research advisory board. This group will serve as the governing body of the Center and will be selected by the Center’s voting membership. The voting membership will be made up of representatives from each specialty area of CIH (which can be defined by a profession, by a therapy or therapeutic approach, and/or by a condition). This group will choose on a decision-by-decision basis whether to put questions out to their broader constituents and will help ensure that the professions have a say in the research ideas and questions pursued by the Center. The approximately 10–12 person CIH Research Advisory Board will help guide research toward overall CIH policy goals.
3. RAND CIH interest group. RAND supports a number of internal interest groups in which researchers share their experiences and discuss various topics including new methodologic approaches. A CIH interest group has already been formed at RAND and will provide the Center with access to RAND researchers who are interested in applying their skills and experience to CIH, and who can also give constructive feedback on research ideas.
4. CIH researcher training. This component of the Center has two main elements. First, the Center could provide a research training home for individuals applying for NCCIH training grants, including the possibility of a T32 institution-based training grant being housed at RAND. Second, RAND already has in place a series of research training programs, some of which are available online or through distance learning such as the NIH Proposal Bootcamp, and others which require travel to the RAND Santa Monica campus such as the RAND Summer Institute.
5. CIH RAND Partnership for research. The purpose of the Partnership is to create a forum by which RAND can assist CIH Institutions in building their research capacity through collaboration with RAND, other research centers of excellence, and with each other to enable them to successfully compete for research funding. The partnership’s primary goal is to develop research capacity by developing fundable research proposals, and this will be achieved through obtaining access to and commitment from a wide variety of mentors. While researchers may partner with RAND itself in their proposals, that will not be required.

### Goals of a collaborative CIH research Center

There are four main objectives of this Center.

A. Provide a platform for collaboration. At present CIH researchers are located across a variety of institutions, sometimes as the sole CIH practitioner or sole CIH researcher, but oft-times without access to the research community required to successfully design, fund and conduct CIH-relevant studies. The CIH Research Network is proposed to address this need.
B. Provide a mechanism by which CIH researchers can stay at CIH institutions. As discussed above, current NIH researcher training usually results in CIH researchers moving away from their institutions. Also, many CIH researchers who stay in their institutions are isolated from their peers and the CIH research community and lack the research infrastructure needed for successful funding. However, if they leave their institutions the research culture for the training of future practitioners is lost. We propose that one criterion for voting membership or a position on the CIH Research Advisory Board be that CIH institutions are required to create “protected time” for their staff for research. This protected time would be minimal on an ongoing basis, but would increase as needed if the researcher is involved in developing a proposal that the membership and/or the Board has voted as ready and worthy to move forward in the Center. Of course, if the researcher is on a grant that is funded their time on the project will be covered by the grant. The CIH Research Network will also benefit and support CIH researchers staying in their institutions.
C. Provide guidance and support to improve the quality of CIH research. All ideas, proposals, and research studies benefit from the input of others with experience in the topic under study. The CIH professions also benefit from research targeting their questions. The CIH Research Advisory Board will help guide and support high-quality CIH research, the CIH Research Network will help support this research, the RAND CIH Interest Group can provide researchers with access to specialty skills and feedback on ideas, and the CIH RAND Partnership for Research can provide mentors and a support team to ensure high-quality proposals and research.
D. Train CIH researchers. As noted above NIH offers a number of training grants. The Center could be one place that that training could be offered. In addition, many others who do not need or want the full immersive experience of a NIH training grant may benefit from shorter term or more targeted research training. The CIH Researcher Training component addresses this goal.

### Next steps – support needed

We have received remarkably enthusiastic and positive feedback from the CIH research community as to the need for this Center and to our basic plan. The third and fourth components of the Center are in place and funded (the RAND CIH Interest Group) or can be put in place as needed and funded through fees (CIH Researcher Training). We will seek philanthropic support but to achieve that we probably need to obtain CIH institution buy-in before we begin. We feel that to be successful we will require support from outside the professional institutions given that a large part of the problem arises from lack of resources to support in-house research infrastructures.

#### CIH institution buy-in meeting

Keeping CIH researchers in their institutions requires the buy-in of those institutions’ leaders. These institutions have to be willing to at least partially support their researchers’ time to participate. To determine their interest and to work out the details of this support—e.g., whether voting power on the CIH Research Advisory Board will be attractive and/or sufficient—we propose to start with an email-based inquiry and subsequent meeting of interested heads of CIH institutions. As noted earlier we are planning our initial scoping meeting with the leaders in September of 2019. The future timeline will be determined by the response of the leaders but also on finding a funding agency. Our intent is to find a funding source that is not the colleges themselves. To date the philanthropic institutions we have spoken to would like to see the initial meeting occur first.

#### CIH Research Network

To build and manage this website it is estimated that we would need about three years for development.

#### CIH Research Advisory Board

Support for this Board and its activities will include travel and meeting costs for in-person meetings at RAND at least one time per year, video conferencing or Adobe Connect costs for long-distance meetings, conference costs for tables/meetings, PR costs for brochures/newsletters, and funding to cover Board members’ time in mentoring researchers and reviewing proposals and ideas.

#### CIH RAND Partnership for Research

This component requires ongoing, secure funding to support the participation of experienced, successful research mentors.

## Conclusion

In analyzing the situation, it is clear that if the institutions pool their limited talent and resources they might be able to compete, but history has shown that the American chiropractic institutions have not yet been able to do that. However, perhaps through a mediator it might be achievable. We are not suggesting this proposal will solve all the problems and we recognize that outside of the US the situation is different. But within the US it seems to us that without some type of response the situation will get worse not better. The RAND Health program has signed onto this proposal and we are currently approaching foundations to fund it.

## Abbreviations

CIH: Complementary and Integrative Health
NCCIH: National Center for Complementary and Integrative Health
ND: Naturopathic doctor
NIH: National Institute of Health
PhD: Doctor of Philosophy

## Authors’ information

IDC is a Senior Health Policy Researcher at RAND and holder of the RAND/Samueli Chair for Integrative Medicine; a Professor in the School of Dentistry UCLA; adjunct faculty member at SCHUS.

PMH is a licensed naturopathic doctor and a Senior Health Policy Researcher at RAND.
